# Ablation of Ghrelin O-Acyltransferase Does Not Improve Glucose Intolerance or Body Adiposity in Mice on a Leptin-Deficient ob/ob Background

**DOI:** 10.1371/journal.pone.0061822

**Published:** 2013-04-22

**Authors:** Henriette Kirchner, Kristy M. Heppner, Jenna Holland, Dhiraj Kabra, Matthias H. Tschöp, Paul T. Pfluger

**Affiliations:** 1 Department of Molecular Medicine and Surgery, Karolinska Institutet, Stockholm, Sweden; 2 Metabolic Diseases Institute, University of Cincinnati, Cincinnati, Ohio, United States of America; 3 Institute for Diabetes and Obesity, Helmholtz Centre Munich, Neuherberg, Germany; University of Bremen, Germany

## Abstract

Type 2 Diabetes is a global health burden and based on current estimates will become an even larger problem in the future. Developing new strategies to prevent and treat diabetes is a scientific challenge of high priority. The stomach hormone ghrelin has been associated with playing a role in the regulation of glucose homeostasis. However, its precise mechanism and impact on whole glucose metabolism remains to be elucidated. This study aims to clarify the role of the two ghrelin isoforms acyl- and desacyl ghrelin in regulating glucose homeostasis. Therefore ghrelin activating enzyme Ghrelin-*O*-acyltransferase (GOAT) was ablated in leptin-deficient ob/ob mice to study whether specific acyl ghrelin deficiency or desacyl ghrelin abundance modifies glucose tolerance on a massively obese background. As targeted deletion of acyl ghrelin does not improve glucose homeostasis in our GOAT-ob/ob mouse model we conclude that neither acyl ghrelin nor the increased ratio of desacyl/acyl ghrelin is crucial for controlling glucose homeostasis in the here presented model of massive obesity induced by leptin deficiency.

## Introduction

Diabetes is one of the major public health burdens affecting industrialized nations and based on current estimates it will become an even larger problem in the future. The development of new strategies to prevent and treat diabetes is thus a scientific challenge of highest priority. However, the precise mechanisms of type 2 diabetes mellitus (T2DM) pathogenesis are still not completely understood. Current efforts therefore aim to identify the key components that mediate insulin sensitivity and glucose homeostasis (reviewed in [1]). Ghrelin, a stomach hormone linked to body weight regulation and obesity, has recently been suggested to be such a key regulator of glucose metabolism [2], [3], [4], [5], [6].

One of the major challenges in understanding ghrelin’s role in glucose metabolism relates to its unique molecular structure. Endogenous ghrelin exists in two principal forms, as a pure 28-amino acid peptide (desacyl ghrelin) and as an acylated peptide (acyl ghrelin) that carries a fatty acid side chain. This side chain originates from medium-chain-fatty-acids and is posttranslationally attached to ghrelin by the recently discovered enzyme ghrelin O-acyltransferase (GOAT) [7], [8]. Currently, there is only one ghrelin receptor (GHSR) identified, and the activation of this receptor requires the presence of the acyl side chain on the ghrelin molecule [9]. Acyl ghrelin increases food intake and adiposity [10] and is the only known orexigenic gastrointestinal hormone. For desacyl ghrelin, to date no receptor has been identified, and pharmacological infusion of desacyl ghrelin has no impact on body weight [11]. Nevertheless, two reports on transgenic desacyl ghrelin overexpressing mice suggest a role for desacyl ghrelin in the regulation of body weight, either by reducing growth [12] or by impairing white adipose tissue development [6]. Central infusion of desacyl ghrelin in rats and mice was further shown to increase food intake in a GHSR-independent manner [13]. Nevertheless, while the role of acyl ghrelin on food intake and body adiposity is well established, a potential impact of desacyl ghrelin on body weight control remains largely uncertain.

Even more conflicting are the effects of both ghrelin isoforms on glucose homeostasis. Glucose tolerance tests in rodents and humans show that systemic infusion of acyl ghrelin impairs glucose homeostasis [14], [15], [16]. Systemic acyl ghrelin administration in mice significantly elevates blood glucose, which can be blocked by co-administration of GHSR antagonists [16]. In contrast, earlier studies in rats suggest that ghrelin decreases blood glucose by increasing insulin release [17], [18]. Desacyl ghrelin was shown to either have no effect on glucose homeostasis in mice [16] and humans [19], or to have beneficial effects on insulin sensitivity [20] and secretion [21], [22]. Some reports further suggest that desacyl ghrelin might oppose acyl ghrelin-mediated glucose regulation [20], [23], [24], [25], e.g. by improving insulin sensitivity [26] or decreasing hepatic glucose output [27]. Concomitantly, data on mouse mutants lacking total ghrelin or acyl-ghrelin are also conflicting. A recent report showed that young animals lacking the expression of GOAT have impaired ability to maintain glycemia during prolonged negative energy balance [2]. Such impairment was prevented when mice were treated with acyl ghrelin, the only known product of GOAT activity. In contrast, we found that calorie restriction does not induce hypoglycemia in 9-month-old mice with ghrelin, GOAT or GHSR deficiency, suggesting that a potential prevention from hypoglycemia may be age-dependent. Total-ghrelin ablation in leptin deficient ob/ob mice was shown to improve glucose homeostasis without altering the body weight [3]. Overall, the conflicting data demonstrate that further research is clearly required to ultimately unravel potential roles of both ghrelin isoforms in glucose control.

In this manuscript, we aim to test the hypothesis that acyl ghrelin deficiency protects from glucose intolerance and body adiposity. Acyl ghrelin deficiency in glucose intolerant and massively obese mice on a leptin deficient background (GOAT-ob/ob) was induced by using the GOAT knockout (GOAT-KO) mouse model. GOAT-KO mice have increased plasma concentrations of desacyl ghrelin but no circulating acyl-ghrelin [7], [28], which makes them an ideal tool to study the actions of desacyl ghrelin when it is unopposed by acyl ghrelin.

## Methods

### Animals

All studies were approved by and performed according to the guidelines of the Institutional Animal Care and Use Committee of the University of Cincinnati. All mice were group housed in Positive Individual Ventilation cages in dedicated animal housing rooms with a 12-h light, 12-h dark cycle (6 am–6 pm) at 22°C and maintained on a standard chow diet (Harlan Teklad LM-485) with free access to food and water, unless indicated otherwise. We further exposed mice for up to 8 weeks to a diet enriched with medium chain triglycerides [28] (MCT diet, Harlan Teklad TD.08622) containing 10% of calories from trioctanoate and tridecanoate.

GOAT-KO mice were generated as previously described [7], [28]. Female Ob/ob mice from Taconic Laboratories were bred with male GOAT^−/−^ mice to generate a double heterozygous GOAT^+/−^OB/ob -F1 generation. This F1 generation was inbred to create mice that were either homozygous leptin deficient (ob/ob) or deficient for both, GOAT and leptin (GOAT-ob/ob mice). For genotyping DNA was extracted by the Genetic Variation and Gene Discovery Core Facility of the Cincinnati Children’s Hospital from tail snips. PCRs to establish the genotype were performed using the primers and thermal conditions described in table 1. All primers were purchased from Integrated DNA Technologies, Inc. in lyophilized form. The genotype of ob/ob mice could further be confirmed phenotypically by the development of obesity 6 weeks after birth.

**Table 1:** Genotyping conditions.

Allele	Primer	PCR conditions	Product
Mboat4^+/+^ Fwd	5′-GGATGGATAAACCTGATGGC-3′	5 min initial 95C, 30 sec 95C, 30 sec 60C, 60 sec 72 sec, 35 cycles, final extension 10 min 72C	238 bp
Mboat4^+/+^ Rev	5′-GCTAAGAGTTCTATATCCAGATCG-3′		
Mboat4^−/−^ Fwd	5′-GCTTAGGGACTCTAGGAAGG-3′	5 min initial 95C, 30 sec 95C, 30 sec 60C, 60 sec 72 sec, 35 cycles, final extension 10 min 72C	277 bp
Mboat4^−/−^ Rev	5′- GCTAAGAGTTCTATATCCAGATCG-3′		
OB/OB and ob/ob Fwd	5'- TGTCCAAGATGGGACCAGACTC-3′	3 min initial 94C, 30 sec 94C, 60 sec 62C, 45 sec 72 sec, 36 cycles, final extension 2 min 72C; Digest product with DdeI at 37C for 7 hrs	OB/OB: 155 bp; ob/ob: 55 and 100 bp
OB/OB and ob/ob Rev	5′-ACTGGTCTGAGGCAGGGAGCA-3′		

Fwd, forward; Rev, reverse; bp, base pairs; Mboat4, Membrane bound-O-acyl transferase 4; ob, obese.

### Glucose Tolerance and Insulin Tolerance Tests

For the measurements of glucose tolerance and insulin sensitivity, mice were subjected to 6 or 16 hrs of fasting and injected intraperitoneally with 1 or 2 g glucose/kg body weight (20% D-glucose (Sigma) in 0.9% saline) for the GTT, and 0.75 or 1.0 U insulin/kg body weight (Humolog, Lily, Indianapolis, USA) for the ITT, as indicated. Tail blood glucose levels [mg/dL] were measured using a handheld glucometer (TheraSense Freestyle) before (0 min) and at 15, 30, 60 and 120 min after injection.

### Body Composition and Indirect Calorimetry

Body weight (BW) was measured using a laboratory scale (Metzler Toledo). Fat mass (FM) and lean mass (LM) was measured using NMR technology (EchoMRI, Houston, TX). Fat fee mass (FFM) was calculated by subtracting the FM from the BW. Energy intake and energy expenditure (EE), as well as home-cage activity, were studied by using the TSE LabMaster system (TSE Systems Gmbh, Bad Homburg, Germany). The calorimetry system was located in a designated room within the animal housing facility with a 12-h light, 12-h dark cycle and 22°C room temperature. Mice had free access to food and water while in the system. After adaptation to the cages for >12 h, oxygen consumption and carbon dioxide production were measured every 45 min for a total of 76 h to determine the respiratory quotient and energy expenditure. Home-cage locomotor activity was determined using a multidimensional infrared light beam system with beams installed on cage bottom and cage top levels and activity being expressed as beam breaks. Stationary motor activity (fidgeting) was defined as consecutive breaks of one single light beam at cage bottom level, ambulatory movement as breaks of any two different light beams at cage bottom level, and rearing as simultaneous breaks of light beams on both cage bottom and top level.

### Plasma Parameters

Blood was obtained from tail blood after an overnight fast using EDTA-coated Microvette® tubes (Sarstedt, Nuremberg, Germany). To each 1 ml of blood 100µl of antiproteolytic cocktail consisting of 27.17 ml EDTA, 100 mg aprotenin, 22.83 ml saline, 4.35 ml heparin, and 23.5 mg diprotinA was added. Blood was centrifuged at 3000 pm for 15 min at 4°C to obtain plasma. Plasma was kept at 4°C during all preparations or was frozen at −20°C for storage. Total ghrelin levels were measured by a radioimmunoassay (Ghrelin (Rat/Mouse) RIA, Phoenixpeptide, Burlingame, California, USA). Insulin levels were measured by a radioimmunoassay from Linco (Sensitive Rat Insulin RIA, Linco Research, St. Charles, Missouri, USA).

### Statistical Analysis

Statistical analysis was performed using GraphPad Prim, Inc. Software Version 5. All data are represented as mean and standard error of the mean. Differences between phenotypes were assessed using 1-way or 2-way Anova with Bonferroni’s post test.

## Results

**Figure 1:**
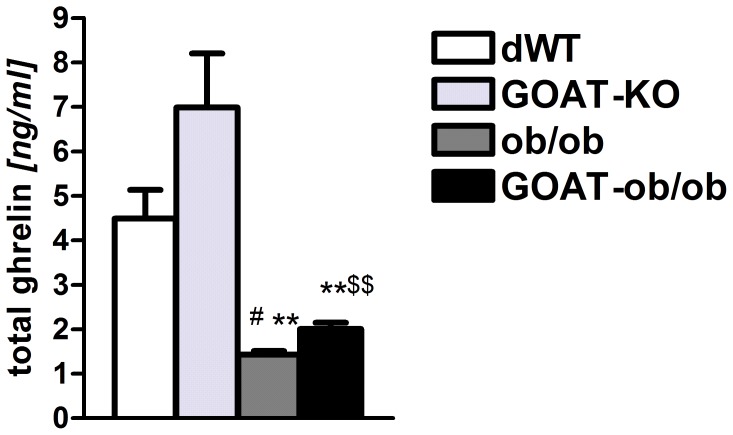
Total ghrelin levels in WT and GOAT-KO mice on a normal or leptin-deficient ob/ob background. GOAT-KO and GOAT-ob/ob mice with a complete lack of acyl ghrelin have increased plasma concentration of total (desacyl) ghrelin. Total ghrelin levels are lower in both ob/ob as well as GOAT-ob/ob mice, compared to lean WT controls or GOAT-KO mice on chow diet. ^#^ P<0.05 versus dWT; ** P<0.001 versus GOAT-KO; ^$$^ P<0.001 versus ob/ob; n = 4–7.

**Figure 2:**
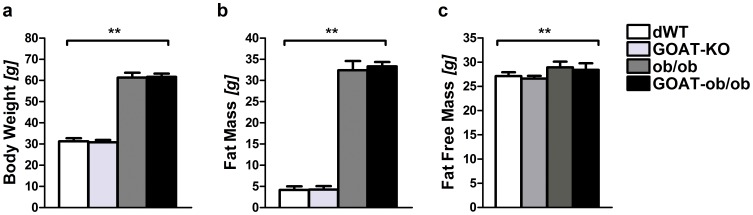
Body weight, fat mass and fat free mass in male mice lacking GOAT on a leptin-deficient ob/ob background. GOAT-ob/ob mice fed standard chow diet display no differences in body weight (a), fat mass (b), or fat free mass (c), compared to ob/ob littermates. However, both leptin-deficient mutants differ significantly in their body adiposity from GOAT-KO and dWT mice, respectively. ** P<0.01 (1-way ANOVA); (n = 5–7).

Leptin-deficient ob/ob mice are morbidly obese and glucose intolerant already at a young age, even when fed with standard chow diet. By co-ablation of GOAT, we aimed to ameliorate both the body adiposity as well as the glucose intolerance phenotype. We first examined whether total ghrelin levels were changed in the chow-fed double-mutant mice. As previously shown GOAT-KO mice have zero acyl ghrelin but increased blood and tissue concentrations of total ghrelin [7]. Similarly, acyl ghrelin was absent from GOAT-KO or GOAT-ob/ob mice (data not shown). GOAT deficiency slightly increased total ghrelin levels regardless of the presence or absence of the ob-allele (Fig. 1). Notably, GOAT-ob and ob/ob mice displayed decreased total ghrelin levels, which can be attributed to their increase in body weight and body adiposity (Fig. 2). However, GOAT ablation in chow-fed ob/ob mice did not improve the morbidly obese phenotype. When fed standard chow there were no differences in body weight and body composition (Fig. 2) between two month old male or female (data not shown) GOAT-ob/ob and ob/ob littermate mice.

**Figure 3:**
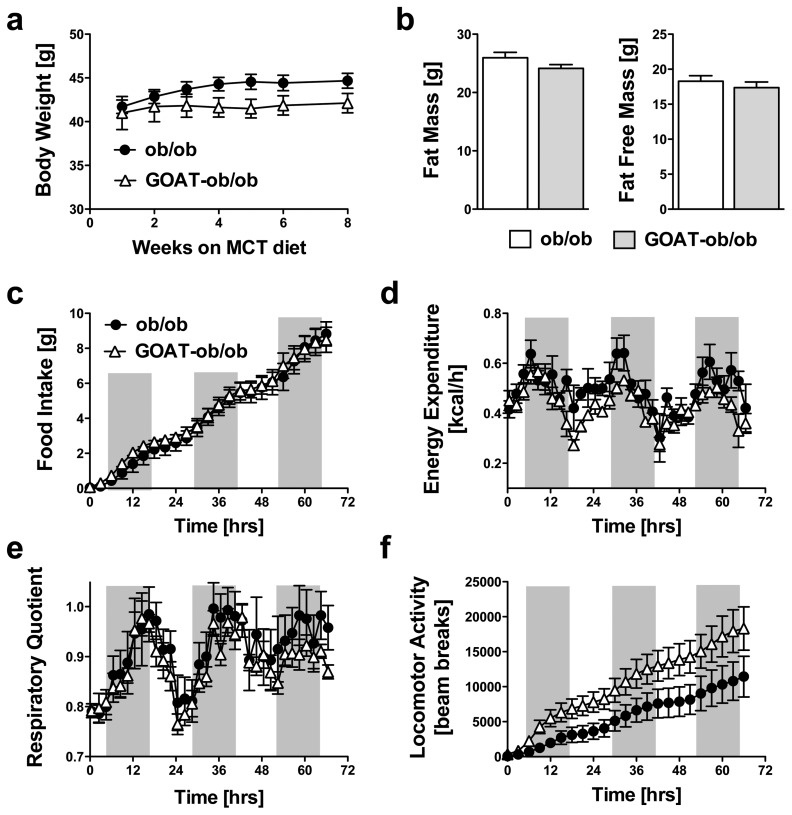
Metabolic phenotypes of ob/ob mutants and GOAT-ob/ob double mutants after exposure to medium-chain triglyceride (MCT) enriched diet. Leptin-deficient ob/ob and GOAT-ob/ob double mutant mice were fed MCT diet for 8 weeks. No differences were observed for body weight (a) and fat mass or fat free mass (b). Further, indirect calorimetry after 7 weeks of MCT diet exposure revealed no differences in food intake (c), energy expenditure (d), respiratory quotients (e) or locomotor activity (f, P = 0.16). (n = 4–7).

Recently, we could reveal that a challenge with a diet enriched in medium chain triglycerides (MCT) can induce profound metabolic alterations in GOAT-KO mice [28]. Therefore, GOAT-ob/ob and ob/ob mice were fed with a diet that contained 10% of calories from medium-chain-triglycerides (MCT diet) at 4 weeks of age. At the time when MCT feeding was started, GOAT-ob/ob and ob/ob mice had similar body weights. GOAT-ob/ob mice developed a persistent trend towards decreased body weight (Fig. 3a), but the difference did not reach the level of statistical significance. GOAT-ob/ob mice further displayed a trend for decreased fat mass and lean mass after 8 weeks of MCT diet feeding (Fig. 3b and c). To study whether the slight differences in body weight and fat mass between GOAT-ob/ob and ob/ob mice were induced by changes in energy expenditure, activity or food intake male mice were placed in an indirect calorimetry system six weeks after the start of MCT feeding. Indirect calorimetry showed that food intake, energy expenditure, and the respiratory quotient were unchanged between both genotypes (Fig. 3c,d,e). Locomotor activity tended to be slightly increased in the GOAT-ob/ob mice but the difference did not reach statistical significance (Fig. 3f).

**Figure 4:**
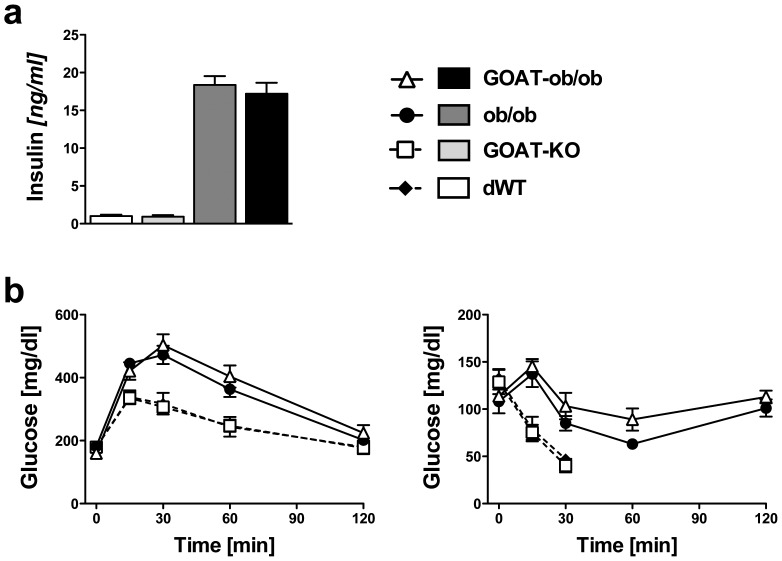
GOAT ablation does not improve glucose homeostasis in mice on a leptin-deficient ob/ob background. a) Fasting insulin levels in 4-month-old chow-fed WT, GOAT-KO, ob/ob mutant and GOAT-ob/ob mice. b) Glucose tolerance tests (b left panel; 1 g glucose/kg body weight) in chow-fed mice revealed no improvement in glucose tolerance by GOAT ablation. Insulin tolerance tests in mice fed with MCT diet (b right panel; 0.75 U insulin/kg body weight) suggested severe insulin resistance in both GOAT-ob/ob and ob/ob mutant mice, and a normal insulin sensitivity in GOAT-KO and dWT mice. (n = 6–10).

Last, we aimed to evaluate whether GOAT ablation could rescue the massive glucose intolerance of leptin-deficient ob/ob mice. We first analyzed fasting insulin levels in chow-fed mice, and saw a massive increase of plasma insulin in both ob/ob as well as GOAT-ob/ob double mutants, compared to the lean double wild-type (dWT) and GOAT-KO mice, respectively. However, we neither saw differences in insulin levels between the lean dWT and GOAT-KO mice, nor between the obese ob/ob mice and double mutants (Fig. 4a). Next, we performed a glucose tolerance test (Fig. 4b), and observed the expected impaired glucose tolerance in ob/ob mice, compared to dWT and GOAT-KO mice. However, GOAT-ob/ob double mutants displayed very similar glucose excursions to ob/ob mutants. Additional glucose tolerance tests in ob/ob mice and GOAT ob/ob double-mutants that were chronically fed with MCT-enriched diet resulted in similar glucose excursions as well (data not shown), corroborating that GOAT ablation does not provide any protection from glucose intolerance in mice on a leptin deficient background. Last, we performed an insulin tolerance test using MCT diet-fed mice (Fig. 4c). Insulin administration (0.75 U/kg) to lean dWT and GOAT-KO mice led to a dramatic decrease in blood glucose. Accordingly, after 30 min both groups were removed from the experiments and rescued from hypoglycemia by administration of glucose. In contrast, both the ob/ob as well as the GOAT ob/ob double mutants remained normoglycemic, and the glucose excursion suggested severe insulin resistance. Although we observed a tendency for slightly improved insulin sensitivity in ob/ob mice compared to the double mutants, 2-Way ANOVA analyses with Bonferonni’s post tests revealed no statistically significant differences.

## Discussion

The gastrointestinal hormone ghrelin is an endogenous regulator of energy homeostasis that potently increases food intake and body adiposity. It may also function as a direct regulator of glucose metabolism as ghrelin has been suggested to have paracrine or autocrine effects on the pancreas, thereby contributing to the regulation of insulin secretion [17]. Recent work has further shown beneficial effects on energy and glucose homeostasis after ablation of the ghrelin acylating enzyme GOAT [2], [28], suggesting that such beneficial effects could be mediated by an increase in the desacyl ghrelin/acyl ghrelin ratio. Sun et al. further reported that the ablation of ghrelin (i.e. deficiency for both acyl and des-acyl ghrelin) could improve the diabetic but not obese phenotype of ob/ob mice [3].

In this study, we aimed to extend these findings and test in a model of GOAT deficiency whether the absence of acyl ghrelin and thus the relative excess of des-acyl ghrelin could have beneficial effects on glucose and energy homeostasis in a model of obesity and glucose intolerance. To test our hypothesis, we generated GOAT-ob/ob double mutant mice and compared their metabolic phenotype to I) ob/ob mutants with functional acyl ghrelin signaling, II) to lean GOAT-deficient mice (with functional leptin signaling) and III) to WT mice. However, in contrast to the results obtained by Sun et al. [24] on ghrelin-ob/ob double mutants, our GOAT-ob/ob double mutants displayed no improvement in glucose homeostasis or body adiposity.

Despite suggestions for a detrimental role of acyl ghrelin in glucose control, our findings show no evidence that selective acyl ghrelin deficiency could rescue the diabetic phenotype of leptin-deficient mice. Accordingly, glucose-modifying actions of desacyl ghrelin when unopposed by acyl-ghrelin could not be verified. In conclusion, we found no evidence in our model that improvement in glucose homeostasis in mice on a leptin deficient background can be mediated by the absence of acyl ghrelin and thus a change in the ratio of acyl/desacyl ghrelin. In addition, our data suggests that desacyl ghrelin may not be a major regulator of glucose or energy homeostasis, at least not in a model of leptin-deficiency with its massive body adiposity and glucose intolerance.

Ghrelin is highly expressed in the pancreas during neonatal development, which implies a crucial role for ghrelin in pancreatic function and development [29]. Ghrelin and its receptor are further expressed in pancreatic islet cells of adult rodents and humans [16], [17], [30]. The controversy, however, originates from whether ghrelin inhibits insulin secretion [14], [31], [32] or enhances insulin secretion [17], [33], [34]. For instance, GHSR-KO mice are protected from diet-induced obesity and glucose intolerance when chronically exposed to high fat diet [35]. Similarly, ghrelin-KO mice demonstrate enhanced glucose homeostasis compared to wild-type littermates after early-onset exposure to HFD by improving glucose tolerance and lowering plasma concentrations of insulin, glucose, leptin, triglycerides, and cholesterol [36]. Moreover, pharmacological inhibition of ghrelin acylation by administration of a peptide-based bisubstrate analog that antagonizes GOAT (GO-CoA-Tat) improves glucose tolerance and reduces weight gain in mice [37]. These data suggest that ghrelin deficiency may protect rodents from HFD-induced hyperglycemia and hyperinsulinemia. If the lack of acyl ghrelin indeed improved glucose homeostasis, a double mutant that lacks leptin and the ghrelin receptor GHSR should display a similar phenotype as ghrelin-ob/ob mutants. However, Ma et al. recently observed that GHSR ablation in leptin deficient ob/ob mice impaired insulin secretion and worsened hyperglycemia [38]. Ma et al. further reported an increase in the pancreatic expression of UCP-2, SREBP-1c, ChREBP, and MIF-1 and a decrease in the expression of HIF-1α, FGF-21, and PDX-1 in their GHSR-ob/ob mutants, which points to a dysregulation of beta-cell function. It further remains possible that GHSR ablation in ob/ob mice could have directly impaired skeletal muscle insulin sensitivity or hepatic glucose control. Overall, these contradicting findings could suggest that a) desacyl ghrelin could mediate detrimental effects on glucose homeostasis via a GHSR independent mechanism, b) acyl ghrelin could have beneficial effects on glucose tolerance or c) that the constitutive activity of the ghrelin receptor is essential to maintain normal glucose control. However, data obtained with our single and double mutant models support neither of these hypotheses; we showed a complete lack of acyl ghrelin and increased desacyl ghrelin levels in our GOAT-ob/ob double mutants. Despite functional GHSR constitutive activity, we observed neither improvement nor impairment in glucose homeostasis compared to ob/ob mutants. Accordingly, the constitutive activity of the ghrelin receptor GHSR does not seem to mediate beneficial effects on pancreatic beta-cell function, at least in the absence of leptin signaling. Nevertheless, further studies and novel models with abolished constitutive activity but an otherwise functional acyl ghrelin signal transduction will be needed to delineate potential detrimental effects of acyl ghrelin signaling from potential beneficial effects of GHSR constitutive activity in the absence of acyl ghrelin. Such studies could also help to explain why earlier reports found improved glucose tolerance in high-fat diet-fed ghrelin-KO [36] and GHSR-KO mice [35] as well as mice treated with a GOAT inhibitor [37].

In summary, the lack of effect in our GOAT-ob/ob double mutants suggests that the ratio of desacyl/acyl ghrelin is not a major denominator for glucose homeostasis in a model of massive obesity and glucose intolerance. Data obtained by us and others on the ablation of all three components of the GOAT-ghrelin-GHSR axis in mice on an ob/ob background further demonstrate that neither desacyl nor acyl ghrelin (signaling) can reverse the massive obesity induced by leptin deficiency. The surprising finding of no effect or improved vs. impaired glucose homeostasis in GOAT-ob/ob, ghrelin-ob/ob and GHSR-ob/ob double mutants further points to a complex and only partially understood role of ghrelin in glucose control. In conclusion, the paradoxical findings obtained by us and others highlight the plurality and complexity of the GOAT-ghrelin-GHSR axis in controlling glucose and energy homeostasis. The discrepant findings between single and double mutants further suggest a close interplay between leptin and ghrelin signaling pathways. They further indicate that impaired leptin signaling can potentially override any beneficial metabolic effects mediated via the GOAT-ghrelin system.
